# Supplementary material for: Retinotopic coding organizes the interaction between internally and externally oriented brain networks
Source: eLife. 2026 Sep 22;15:RP110234. doi: 10.7554/eLife.110234 (PMC13597088; doi:10.7554/eLife.110234)
Supplement: Peer review history

# Editor report: eLife Assessment

**DOI:** 10.7554/eLife.110234.3.sa0

**Authors:** Peter Kok

**Affiliations:** University College London United Kingdom

**Keywords:** Fundamental, Solid

This study addresses a **fundamental** question about how large-scale brain networks interact, and specifically how the default mode network exchanges information with sensory cortex. The analyses provide **solid** evidence for the claims made in the paper. The findings should be of broad interest to researchers studying brain network organization and dynamics.

# Referee report: Reviewer #1 (Public review)

**DOI:** 10.7554/eLife.110234.3.sa1

Summary:

This paper leverages 7T fMRI data from the Natural Scenes Dataset to investigate whether retinotopic coding the position-selective organization of visual responses structures spontaneous resting-state interactions between the Default Network (DN) and the Dorsal Attention Network (dATN). Using individualized network parcellations and population receptive field (pRF) modeling, the authors show that DN voxels can be split into two subpopulations based on their response to visual stimulation: those with position-specific positive BOLD responses (+pRFs) and those with position-specific negative BOLD responses (-pRFs). Critically, these subpopulations relate differently to the dATN during rest: -pRFs are anticorrelated with the dATN, +pRFs are positively correlated, and non-retinotopic DN voxels show no coupling. The anticorrelation (and positive correlation) is enhanced when DN and dATN voxels share visual field preferences. An event-triggered analysis suggests that retinotopic coding shapes both "top-down" (DN-initiated) and "bottom-up" (dATN-initiated) spontaneous activity transients, supporting the claim that the retinotopic scaffold is intrinsic to the DN. These findings challenge the prevailing view of global DN-dATN antagonism and suggest retinotopic coding as an organizing principle for cross-network communication.

Strengths:

The central finding that what looks like network-level independence between DN and dATN decomposes into structured, bivalent interactions organized by voxel-level visual field preferences is a compelling demonstration that macro-scale network descriptions can hide meaningful substructure. The logic of the analysis is clean: pRF properties are estimated from retinotopic mapping data and then used to predict resting-state coupling in completely independent scanning sessions. This cross-session, cross-modality design rules out many circularity concerns.

The use of individualized multi-session hierarchical Bayesian parcellation (Kong et al.) to define DN and dATN boundaries within each subject is the right methodological choice for this question. Network boundaries in posterior cortex, where DN and dATN interdigitate most closely, vary considerably across individuals, and group-average approaches would introduce exactly the kind of misassignment that would most confound the result.

The matched-vs-random pRF analysis is well-controlled. The authors demonstrate that cortical distance between matched and randomly matched dATN pRFs does not differ, effectively ruling out spatial proximity on the cortical surface as a confound. tSNR controls further show that signal quality differences do not drive the effect.

The event-triggered analysis (Figure 3) is creative and adds genuine value. Showing that retinotopically-specific coupling persists during DN-initiated activity transients not only dATN-initiated ones is the key piece of evidence for the claim that the code is intrinsic to the DN rather than passively inherited through bottom-up visual drive.

The result is observed consistently across all individual participants, which provides strong evidence for the robustness of the qualitative pattern despite the small sample size inherent to densely sampled designs.

Comments on revised version:

I'm content with the additional analyses and alterations to the writing that the authors have performed. I'm convinced that this work will spawn a very productive thread in the literature.

# Referee report: Reviewer #2 (Public review)

**DOI:** 10.7554/eLife.110234.3.sa2

Summary:

Using a public dataset of retinotopic mapping and resting-state data, the authors find that the default mode network has voxels that respond (positively or negatively) to visual stimulation at specific retinotopic positions, and that resting-state activity in these voxels is correlated with activity in more traditional sensory voxels with the same visual-location preference. The retinotopic specificity is bidirectional, such that high activity in default mode voxels drives activity only in voxels with matching receptive fields in sensory cortex, and vice versa. These findings are at odds with traditional views of the default mode network as having abstract (non-retinotopic) representations and competing (rather than cooperating) with external sensory representations.

Strengths:

This study continues an intriguing line of research about how default mode regions interact with sensory cortex. Demonstrating that there are structured interactions between these regions at rest, and that these interactions are in fact organized according to retinotopic location (as opposed to traditional views of representational format in the default mode network), provides a new framework for thinking about large-scale internal and external brain networks. The authors make use of a well-powered public dataset that allows for precise estimates of pRFs and individual-specific resting-state networks and develop a number of interesting analyses that characterize the relationships between DN and dATN voxels. The findings are exciting and could have a major impact on future studies in cognitive neuroimaging.

The authors mention that these findings could shed light on internal/external interactions such as "anticipatory saccades or memory-guided attention," which is true, though I would argue that constructing DN representations of external stimuli is in fact even more fundamental than these specific cases (e.g. see Barnett and Bellana, 2025, "Situation models and the default mode network"). The "highways" identified in this study could play a vital role in real-world perceptual processes that are constantly translating external input into internal mental models.

Weaknesses:

(1) The criterion used for defining voxels as retinotopic seems very liberal. The authors show that only 5% of voxels have R^2>0.14 in a null analysis and therefore define voxels with R^2>0.14 as retinotopic. Although all the networks in Fig 1C show voxel distributions that differ from the null, the number of false positives above R^2>0.14 seems problematic, especially for the DN positive pRFs (red distribution) and to a lesser extent the DN negative pRFs (blue distribution). From visual inspection of the plot, the false discovery rate (fraction of voxels labeled as retinotopic that are false positives) looks like it would be greater than 50% for the DN positive pRFs. The authors do show that the positive pRF voxels have above-chance consistency across runs and also show in a supplementary analysis (Fig S5) that applying a stricter R^2 criterion yields similar results. These help to mitigate this concern, providing evidence that there are true positive voxels in this set which are driving the effects.

(2) The claim that "voxel-level visual response profiles shape DN-dATN coupling during spontaneous resting-state activity" is well-supported for specific sub-groups of DN voxels, though it is unclear whether the overall DN-dATN correlation at rest is primarily driven by the pRF-tuned voxels investigated in this study.

(3) The event-triggered analysis is effective at testing the bidirectional relationship between DN and dATN, with high activity in either network triggering a response in the other network. However, it would be helpful to show more validation that these "events" are meaningful windows of time to study, and that 13 TRs a typical length of time that activity is elevated during one of these events.

(4) The framing of this paper relative to the authors past work, such as Steel et al. 2024 ("A retinotopic code structures the interaction between perception and memory systems") could be improved. The primary novelty here is that this paper examines resting-state data and individually defined whole-brain networks, showing that there are widespread spontaneous interactions between broad internal and external networks, but this distinction is not made explicit in the Introduction.

# Referee report: Reviewer #3 (Public review)

**DOI:** 10.7554/eLife.110234.3.sa3

Summary:

This paper addresses an important question (relationship between DN and dATN, and the role of retinotopic coding) and uses a set of novel analyses.

Strengths:

Important question, novel analytical approaches (pRF-informed functional connectivity analysis).

Weaknesses:

Some of the analyses are not described with sufficient clarity, especially the final analysis related to Fig. 3.

Comments on revised version.

Related to my previous comment (3), the removal of the labels "bottom-up" and "top-down" in the final analysis is a big improvement. However, I still don't fully understand how the 10 most aligned pRFs and the 10 most anti-matched pRFs are selected. The methods section on this has some ambiguity: "the 10 with the smallest Euclidean distance in RF center (x,y)". Does this mean that these are the pRFs closest to fovea? If not, what is the Euclidean distance referring to? Likewise, I don't understand how the anti-matched voxels are selected. This makes the interpretation of Fig 3 difficult.

My previous comment about baseline activation was to compare the matched voxels with randomly selected voxels, instead of with anti-matched voxels. The authors responded that there was a technical difficulty with this.

# Author comment: Author response

**DOI:** 10.7554/eLife.110234.3.sa4

**Authors:** Adam Steel, Peter A Angeli, Caroline Robertson

**Affiliations:** Dartmouth College Hanover United States; Dartmouth College Hanover United States

The following is the authors’ response to the original reviews.

**Public Reviews:**

**Reviewer #1 (Public review):**

Summary:

This paper leverages 7T fMRI data from the Natural Scenes Dataset to investigate whether retinotopic coding, the position-selective organization of visual response structures, spontaneous resting-state interactions between the Default Network (DN) and the Dorsal Attention Network (dATN). Using individualized network parcellations and population receptive field (pRF) modeling, the authors show that DN voxels can be split into two subpopulations based on their response to visual stimulation: those with position-specific positive BOLD responses (+pRFs) and those with position-specific negative BOLD responses (-pRFs). Critically, these subpopulations relate differently to the dATN during rest: -pRFs are anticorrelated with the dATN, +pRFs are positively correlated, and non-retinotopic DN voxels show no coupling. The anticorrelation (and positive correlation) is enhanced when DN and dATN voxels share visual field preferences. An eventtriggered analysis suggests that retinotopic coding shapes both "top-down" (DNinitiated) and "bottom-up" (dATN-initiated) spontaneous activity transients, supporting the claim that the retinotopic scaffold is intrinsic to the DN. These findings challenge the prevailing view of global DN-dATN antagonism and suggest retinotopic coding as an organizing principle for cross-network communication.

Strengths:

The central finding that what looks like network-level independence between DN and dATN decomposes into structured, bivalent interactions organized by voxellevel visual field preferences is a compelling demonstration that macro-scale network descriptions can hide meaningful substructure. The logic of the analysis is clean: pRF properties are estimated from retinotopic mapping data and then used to predict resting-state coupling in completely independent scanning sessions. This cross-session, cross-modality design rules out many circularity concerns.

The use of individualized multi-session hierarchical Bayesian parcellation (Kong et al.) to define DN and dATN boundaries within each subject is the right methodological choice for this question. Network boundaries in posterior cortex, where DN and dATN interdigitate most closely, vary considerably across individuals, and group-average approaches would introduce exactly the kind of misassignment that would most confound the result.

The matched-vs-random pRF analysis is well-controlled. The authors demonstrate that cortical distance between matched and randomly-matched dATN pRFs does not differ, effectively ruling out spatial proximity on the cortical surface as a confound. tSNR controls further show that signal quality differences do not drive the effect.

The event-triggered analysis (Figure 3) is creative and adds genuine value. Showing that retinotopically-specific coupling persists during DN-initiated activity transients, not only dATN-initiated ones, is the key piece of evidence for the claim that the code is intrinsic to the DN rather than passively inherited through bottom-up visual drive.

The result is observed consistently across all individual participants, which provides strong evidence for the robustness of the qualitative pattern despite the small sample size inherent to densely-sampled designs.

Weaknesses

(1) The nature of negative pRFs requires more scrutiny

The entire interpretive framework depends on treating negative pRFs in the DN as genuine position-selective neural responses (suppression). However, negative BOLD signals are well known to arise from non-neural sources, specifically, vascular stealing (where activation in nearby tissue diverts blood from adjacent voxels) and macrovascular draining vein effects that produce spatially displaced signal inversions. These concerns are amplified at 7T, where T2*-weighted GEEPI carries substantial macrovascular weighting. The DN and dATN interdigitate extensively in the posterior cortex, often within millimeters. A negative pRF in a DN voxel adjacent to a positive dATN voxel could, in principle, reflect the hemodynamic shadow of its neighbor rather than an independent neural response.

The spatial dispersion control (matched vs. random pRFs have similar cortical distribution) is valuable but addresses long-range confounds, not local hemodynamic crosstalk. The reliability of sign and center position across runs is reassuring but does not exclude a vascular origin, as vascular architecture is itself stable across sessions. I would encourage the authors to test whether the matched-vs-random effect survives exclusion of voxels near large pial vessels (identifiable from T2* contrast or the venograms available in the NSD). These analyses would not be dispositive, but they would meaningfully strengthen the neural interpretation.

The reviewer raises an important concern about the interpretation of negative pRFs in the DN, namely that spatially specific negative BOLD responses could, in principle, reflect local vascular effects rather than genuine position-selective suppression. The reviewer suggests excluding voxels near large vessels to address this issue.

Based on the reviewer’s suggestion, we repeated the pRF matching analysis excluding any voxels within 3mm of a major vein, as identified using the time-of-flight (TOF) MR venography included in the NSD. This analysis therefore tests whether the retinotopically specific DN–dATN coupling persists after removing voxels most likely to be affected by vascular signal.

Excluding these voxels did not impact our results: we found preferential coupling according to response valence and center position, with stronger correlation between matched +DN and +dATN voxels (t(6) = 6.054, *p* < 0.001), and a more pronounced negative correlation between matched -DN and -dATN voxels (t(6) = -5.0448, *p* < 0.01). We have added these results to the supplemental figures (Fig. S7), and also added to the text (Pg. 8). Together with the run-wise reliability of pRF sign and position, and the persistence of the matched-versus-random effect after vessel exclusion, this analysis supports the interpretation that negative DN pRFs reflect structured, spatially specific responses rather than a vascular artifact.

“Finally, to rule out any possible influences from vascular stealing (i.e. the shunting of blood into active tissue from nearby regions), we repeated the matching analysis after excluding any voxels within a 3mm radius of a major vessel (Fig. S7; see Methods). Both matching effects remained after excluding vascularly susceptible voxels (+DN x dATN: t(6) = 6.054; p < 0.001; -DN x dATN: t(6) = -5.0448; p < 0.01).”

(2) Amount of retinotopic mapping data and choice of pRF pipeline

The NSD includes 6 runs of retinotopic mapping (~5 minutes each; 3 baraperture, 3 wedge/ring). The authors use only the 3 bar-aperture runs (~15 minutes total per subject) and fit their own pRFs using AFNI's 3dNLfim procedure, rather than using the pRF estimates provided as part of the NSD release (which were fitted using the analyzePRF toolbox with all 6 runs).

Fifteen minutes of bar data is quite limited for reliable voxel-wise pRF estimation, especially in regions far from the early visual cortex, where signal-to-noise is inherently lower. Standard recommendations for robust pRF mapping in higherorder regions generally suggest substantially more data. The variance-explained threshold is close to the noise floor by design, meaning that a non-trivial number of the "retinotopic" DN voxels may be poorly estimated. Given that the core analyses depend on both the sign and the center position of these pRFs, the limited data is a significant concern.

The authors do not explain why they chose to re-fit pRFs rather than use the NSD-provided estimates. If the motivation was methodological (e.g., the NSD pRF pipeline does not readily yield signed amplitude, or the bar-only fits were judged more appropriate for detecting negative responses), this should be made explicit. If the NSD-provided pRFs can reproduce the key findings, this would substantially increase confidence in the results. If they cannot, that divergence itself would be important to understand. I would ask the authors to address this choice and, if feasible, to report whether the core results replicate using the NSDprovided pRF estimates and/or whether using all 6 runs of retinotopy data changes the findings.

The reviewer raises two related concerns: first, that the amount of retinotopic mapping data available in the NSD may be limited for estimating voxel-wise pRFs in higher-order cortical regions; and second, that we re-fit the pRF model using AFNI rather than relying on the pRF estimates provided with the NSD release. We appreciate the opportunity to clarify both points. We agree with the reviewer that more travelling bar data would be preferable and would likely yield more robust model fits, particularly in higher-order regions with lower SNR. This is a limitation of our paper that we now acknowledge in the discussion section. However, we do not think that more data would fundamentally change the pattern of our results for the following reasons.

First, we implemented a novel data-driven approach to derive a threshold for thresholding significant pRF fits (a noise floor). Importantly, our noise floor estimation yields a conservative threshold (R^2^ > 0.14), which is greater than both our previous work characterizing cortical pRFs (Steel et al. 2024: R^2^ > 0.08) and other work exploring visual responses in the default network (Klink et al. 2021: R^2^ > 0.05; no threshold: Szinte and Knapen 2020; Knapen 2021).

Second, the key pRF features used in our analyses – response sign and centre position – were reliable across retinotopic mapping runs. This reliability is important because our central matching analysis depends on voxel-wise estimates of both response valence and visual-field position.

Third, the matching analysis asks whether pRF parameters estimated from the retinotopic mapping task predict functional coupling measured during independent resting-state scans. Noisy or unstable pRF estimates should weaken this relationship, because they would degrade the accuracy of voxel-wise matching. Thus, parameter instability would be expected to obscure retinotopically specific coupling rather than systematically produce the observed matched-versus-random effects.

To the reviewer’s question about our decision to re-fit the pRF model using AFNI, the reviewer is correct that this was motivated by the requirements of our analysis: we re-fit the pRF estimates using AFNI because it allows for both positive and negative signed amplitudes. The pRF model fits provided with the NSD do not allow bivalent amplitude estimates. We have made this decision clearer in the text, reproduced below (Pg. 4-5; Pg. 14-15).

“We chose to re-fit the data using a simple Gaussian approach as implemented in AFNI to allow for both positive and negative signed amplitudes.”

“The limited amount of pRF mapping task data included in the NSD posed a challenge for establishing reliable visual response estimates. Here, we addressed this issue by developing a novel thresholding method to establish robust voxel-wise model fits. Among voxels that passed this empirical threshold, we observed a significant correlation in voxel-wise estimates of centre position and visual response amplitude. In addition, our pRF matching results were based on the relationship between the voxel-wise estimates of centre position and response amplitude with resting-state fMRI – a completely independent measure. Crucially, noisy estimates of pRF parameters would obscure this relationship and make our results less likely. Therefore, despite the relatively limited pRF mapping data available, unstable pRF estimates are unlikely to drive our results.”

(3) pRF model adequacy for the Default Network

The isotropic Gaussian pRF model was developed for and validated in early and mid-level visual cortex, where it captures the dominant spatial selectivity of neuronal populations. In DN voxels where the model explains comparatively little variance, it is less clear that the model is capturing the right quantity.

Specifically, the negative pRFs could conceivably be described by a model with a dominant suppressive surround (e.g., a difference-of-Gaussians model), in which what appears as a "negative pRF" in the standard model is actually the surround component of a center-surround mechanism whose center is poorly resolved. This distinction matters: a genuine inverted code (negative center response) implies a qualitatively different computation than inherited surround suppression from nearby visual cortex.

The authors should consider discussing why the standard model is sufficient for the questions asked, or ideally, testing whether the sign distinction survives under alternative pRF model specifications.

We appreciate the reviewer’s comment about the limitations of a single gaussian pRF model. We chose the single gaussian model as a direct extension of prior work from our lab and others (Steel et al., 2024, Klink et al., 2022, Szinte and Knapen, 2021). We agree that a negative response in this model could, in principle, reflect a more complex spatial profile, such as a dominant suppressive surround. However, adjudicating among alternative pRF models would require more retinotopic mapping data than are available in the NSD, particularly for higher-order cortex. Thus, we feel that it is outside the scope of the current work. We now address this limitation in our discussion (Pg. 15).

“Relatedly, here we used a single gaussian model, consistent with prior work on negative visual responses in memory systems (31, 33, 34). However, other models of visual receptive fields might offer further insight into the DN’s visual responsiveness, such as double gaussian models of surround suppression (65) or compressive summation (66). Future studies might directly compare different visual models to further refine the computations underpinning visual responses in the DN.”

(4) Interpreting resting-state transients as top-down vs. bottom-up The event-triggered analysis labels high-amplitude DN pRF activations as "topdown events" and dATN activations as "bottom-up events." This is a reasonable inference given experience-sampling studies showing that rest involves alternation between internal and external attention, but it remains an inference. Without concurrent experience sampling, eye-tracking, or physiological monitoring, we cannot establish that a spontaneous DN transient reflects memory retrieval or internally-directed thought rather than a global arousal fluctuation. Similarly, dATN transients during rest could reflect covert shifts of spatial attention to remembered or imagined locations rather than bottom-up processing per se. I would ask the authors to soften this framing or to discuss what additional data would be needed to validate the top-down/bottom-up attribution.

The reviewer raises an important concern about the strong interpretation of elevated BOLD activity detected in the DN and dATN as top-down and bottom-up events. We agree that the limitations of fMRI in our current data prevent these strong claims about the origin of these signals. We have therefore softened this framing throughout the manuscript, and we now refer to these events as DN-driven and dATN-driven. We think that this more directly describes the analysis: events were defined by transient high-amplitude activity in DN or dATN pRFs, respectively.

(5) The "retinotopic code" vs. "visual field bias" distinction The paper uses the language of a "retinotopic code" throughout and correctly distinguishes this from a "retinotopic map," noting that DN voxels do not form a continuous topographic representation on the cortical surface. This distinction deserves greater emphasis. In vision science, retinotopic maps carry computational significance through their topographic continuity and relationship to cortical wiring. A distributed collection of voxels with coarse visual field preferences but no cortical topography is a fundamentally different organizational feature. Recent reviews have drawn an explicit distinction between retinotopic maps and visual field biases (Groen, Dekker, Knapen & Silson, TiCS 2022), and the present findings may be more accurately characterized as the latter. Perhaps the authors think that the distinction is merely a signal-to-noise distinction, in which case I would invite them to clearly speak to this interpretation. In any case, this is not a criticism of the findings themselves, but clarity on this point would prevent conflation of two different organizational principles and would help position the work for both the vision and network neuroscience communities.

The reviewer raises a valuable point about the distinction between a retinotopic code, a retinotopic map, and a visual field bias, and we are happy to add discussion of this topic to our manuscript.

Our results show that the DN does not exhibit a continuous retinotopic map in the sense used in early visual cortex. Rather, our results suggest a distributed voxel-level code for visual-field position: individual DN voxels show reliable spatial preferences, and these preferences predict retinotopically specific functional coupling with dATN voxels. This voxel-level organization is analogous to other distributed spatial codes, such as head-direction coding in retrosplenial cortex, where spatial variables are represented by population activity without requiring a topographic map on the cortical surface. This differs from a coarse visual-field bias, including preferential responses to the contralateral visual field, although we do also observe such biases. We have added text unpacking this important distinction to the Discussion (Pg. 15-16):

“Prior work has emphasized the visual response bias in regions where voxel-wise retinotopic responses lack a map-like organization(35); overall, the DN does exhibit this kind of bias. However, our results show that the voxel-scale activity underpinning this bias reflects the latent connectivity of those voxels. Thus, we adopt the term “retinotopic coding”, because this voxel-scale coding scheme exists without a map-like organization on the cortical surface. For example, rodent and bat head direction cells are not laid out in a literal ring, but the population code of these neurons forms a ring manifold(68, 69).”

**Reviewer #2 (Public review):**

Summary:

Using a public dataset of retinotopic mapping and resting-state data, the authors find that the default mode network has voxels that respond (positively or negatively) to visual stimulation at specific retinotopic positions, and that restingstate activity in these voxels is correlated with activity in more traditional sensory voxels with the same visual-location preference. The retinotopic specificity is bidirectional, such that high activity in default mode voxels drives activity only in voxels with matching receptive fields in sensory cortex, and vice versa. These findings are at odds with traditional views of the default mode network as having abstract (non-retinotopic) representations and competing (rather than cooperating) with external sensory representations.

Strengths:

This study continues an intriguing line of research about how default mode regions interact with the sensory cortex. Demonstrating that there are structured interactions between these regions at rest, and that these interactions are in fact organized according to retinotopic location (as opposed to traditional views of representational format in the default mode network), provides a new framework for thinking about large-scale internal and external brain networks. The authors make use of a well-powered public dataset that allows for precise estimates of pRFs and individual-specific resting-state networks, and develop a number of interesting analyses that characterize the relationships between DN and dATN voxels. The findings are exciting and could have a major impact on future studies in cognitive neuroimaging.

The authors mention that these findings could shed light on internal/external interactions such as "anticipatory saccades or memory-guided attention," which is true, though I would argue that constructing DN representations of external stimuli is in fact even more fundamental than these specific cases (e.g., see Barnett and Bellana, 2025, "Situation models and the default mode network"). The "highways" identified in this study could play a vital role in real-world perceptual processes that are constantly translating external input into internal mental models.

Weaknesses:

(1) The criterion used for defining voxels as retinotopic seems very liberal. The authors show that only 5% of voxels have R^2>0.14 in a null analysis, and therefore define voxels with R^2>0.14 as retinotopic. Although all the networks in 1C show voxel distributions that differ from the null, the number of false positives above R^2>0.14 seems problematic, especially for the DN positive pRFs (red distribution) and to a lesser extent the DN negative pRFs (blue distribution). From visual inspection of the plot, the false discovery rate (fraction of voxels labeled as retinotopic that are false positives) looks like it would be greater than 50% for the DN-positive pRFs. The authors do show that the positive pRF voxels have abovechance consistency across runs, again providing evidence that there are true positive voxels in this set, but perhaps a stricter criterion (such as having consistent negative fits across runs) would provide more targeted identification of the DN voxels with true retinotopic sensitivity.

We thank the reviewer for giving us the opportunity to discuss this important decision. We agree with the reviewer that a stricter R^2^ criterion could result in more targeted pRF identification. Motivated by the reviewer’s suggestion, we repeated the cross-region pRF matching analysis across multiple R2 thresholds.

The retinotopic matching effects were not dependent on the original threshold. In fact, we found that the pRF matching effects are enhanced as the R^2^ value increases (Fig. S5). This pattern suggests that any false-positive voxels admitted near the original threshold would dilute, rather than drive, the observed matched-versus-random effects. We have added text to the results highlighting this finding (Pg. 7):

“In contrast, DN voxels that responded positively to visual stimulation (DN positive pRFs, +pRFs) had a positive correlation with the dATN (mean correlation = 0.284±0.152, t(6) = 4.96, p = 0.0025), while DN voxels with systematic negative responses to visual stimulation (DN negative pRFs, -pRFs) were anti-correlated with the dATN (mean correlation = -0.21±0.149, t(6) = -3.75, p = 0.0094). This relationship was further strengthened by adopting more conservative R^2^ thresholds up to 0.30 despite the overall number of included voxels decreasing, suggesting that this effect is not driven by false-positive voxels at the edge of our threshold criteria (Fig. S5).”

(2) The claim that "opponency at rest between the DN and dATN appears to be driven by the subset of DN voxels with negative retinotopic tuning" is not well supported. The fraction of DN voxels with negative pRFs is small: 9.42% of DN voxels have pRFs, and 58.77% are negative, so about 6% of DN voxels have negative pRFs. The fact that any DN voxels have negative pRFs is notable, but the authors do not provide evidence that these 6% are driving the overall behavior of the DN. They do show (e.g., in Figure 2B) that negative and positive pRFs have opposing influences, but the overall correlation with dATN does not look similar to the negative pRF connectivity. I'm also unsure whether "opponency" is a reasonable description for two networks that are "independent (i.e., not correlated)" in this analysis.

The reviewer raises an important point about whether negative DN pRFs should be described as driving the overall DN–dATN relationship. We agree that this language was too strong. Negative pRFs constitute a small subset of DN voxels, and our analyses show that this subset has a distinct pattern of functional coupling with the dATN, not that it explains the global relationship between the DN and dATN as a whole.

We have therefore revised the manuscript to avoid implying that negative DN pRFs drive overall DN–dATN opponency. Instead, we now frame these voxels as an important retinotopically tuned subpopulation nested within broader network dynamics. Specifically, our results show that visually responsive DN voxels are not homogeneous: positive and negative DN pRFs show opposing patterns of coupling with dATN pRFs, and these interactions are strengthened when voxels share visual-field preferences. This suggests that a small but structured subset of DN voxels may provide a route for retinotopically specific communication between internally and externally oriented networks, without implying that this subset determines the mean activity pattern of the entire DN:

“Spontaneous DN and dATN activity during rest is uncorrelated at the network level. However, voxel-scale functional coupling across networks is shaped by the latent visual field preferences of individual voxels in each network, as measured during independent retinotopic mapping.” Abstract (Pg. 2)

“This result shows that voxel-level visual response profiles shape DN-dATN coupling during spontaneous resting-state activity. Specifically, the DN and dATN activation is independent during rest. However, at the voxel-level, specific sub-groups of DN voxels have distinct coupling patterns with the dATN that depends on the valence of voxels’ visual responses. DN and dATN voxels with positive visual responses show a positive relationship during rest, and a notable subset of DN voxels with negative visual responses display the canonical opponency with dATN voxels. This suggests that retinotopic coding may be a mechanism that enables visual information to be exchanged between these large-scale brain systems. Specifically, opponency at rest between the DN and dATN appears to be driven by the subset of DN voxels with negative retinotopic tuning.” Results (Pg. 7)

These findings offer a multi-scale account of neural communication, in which interactions among sub-populations of voxels with shared tuning preferences are nested within macro-scale network dynamics. Nesting multiple neural codes might enable ongoing computations within a larger brain system (e.g., attending to internal mental states within the DN during memory recall), while simultaneously allowing for the sharing of fine-grained representations across brain systems (34). Discussion (Pg. 14)

(3) The event-triggered analysis is effective at testing the bidirectional relationship between DN and dATN, with high activity in either network triggering a response in the other network. However, it would be helpful to show more validation that these "events" are meaningful windows of time to study. First, is 13 TRs a typical length of time that activity is elevated during one of these events? Second, the top-down and bottom-up terminology is perhaps too loaded and not well-justified; if the negative pRFs in the DN reflect a meaningful coding system, then couldn't low (rather than high) activity indicate a top-down event?

We thank the reviewer for these helpful suggestions. To the best of our knowledge, there is not currently a widely agreed-upon time window for performing event-based fMRI analyses. We chose a 13 TR time window to balance between sufficiently capturing BOLD signal related to the chosen event while also minimizing influence from other signal fluctuations, based on the procedure adopted in Gordon et al. (Nature, 2023) and Mitra et al. (J. Neuro Phys, 2014), which considered temporal relationships among brain regions over comparable timescales. In our analysis, this window considered 6 TRs (9.6s) on either side of the detected event, which we felt comfortably captures the peak BOLD signal that would result from an impulse at the event time, and responses that may reflect upstream activity leading into it.

The reviewer has raised an additional comment about the terms “top-down” and “bottom-up.” These concerns were shared by Reviewer 1. Based on these comments, we have adopted the terms “DN-driven” and “dATN-driven”, which we think aligns more closely with our analysis approach.

(4) The framing of this paper relative to the authors' past work, such as Steel et al. 2024 ("A retinotopic code structures the interaction between perception and memory systems"), could be improved. The existence of negative pRFs in the DN and a functional relationship between these pRFs and the sensory pRFs have already been described in prior work. My understanding of the primary novelty here is that this paper examines resting-state data, showing that there are widespread spontaneous interactions between broad internal and external networks, but this distinction is not made explicit in the Introduction.

We appreciate the opportunity to clarify the novel aspects of our paper. The reviewer correctly identifies the extent of prior work, which identified -pRFs in regions of the canonical default network (Szinte and Knapen, 2021; Klink et al., 2022) and characterized the local interactions between adjacent perceptual and mnemonic regions (Steel et al., 2024). Our current work builds upon these findings in two key ways.

First, we explore the effect across individually-defined whole brain networks. While the DN and dATN are often adjacent, these networks are spatially discontinuous and are comprised of distinct sub-regions (e.g. in prefrontal cortex). Whether retinotopic patterning of activity would persist in distributed networks could not have been extrapolated from our prior work. We think that finding will be of broad interest to the community studying perception and memory systems, because it offers a mechanistic account of how information is read in/out of memory.

Second, here we considered whether spontaneous activity across networks would be structured by a retinotopic code. Our previous work characterized activity during tasks that depended on visual information: either scene perception or mental imagery. While the prior work was an important first step, it left open the possibility that retinotopic coding may only be relevant in visual tasks. By demonstrating that the retinotopic coding structures voxel-specific coactivation during rest, which entails no overt visual demands, we provide evidence that retinotopic features are a general, mode-agnostic code between regions.

(5) The definition of the default mode (DN) in this study aligns with past research, but the definition of the dorsal attention network (dATN) seems at odds with standard terminology. For example, the authors cite Fox et al. 2006, which depicts the dATN as including regions such as IPS, FEF, SMA, and MT+. Here, however, the "dATN" seems to be primarily lateral and ventral visual cortex (e.g., Figure S5). The exact location of these sensory pRFs is not critical to the authors' claims, but this labeling seems incorrect, and the motivation for defining/selecting the sensory network in this way is not described.

We thank the reviewer for this insightful comment and their careful consideration of our network definition.

Our method for network identification, and the topography of the resulting networks, are broadly consistent with more recent conceptualizations of the DN and dATN (e.g. Du et al. 2024, Gordon et al. 2017, Braga and Buckner 2017). Relatedly, because we defined brain networks based on the unique connectivity patterns of each individual participant, we expect them to differ from previous group-level network descriptions. The increased resolution of the 7T data in the NSD may also result in greater departure from prior definitions compared to previous work done at 3T.

Further study into dATN differences between group-level 3T, individualized 3T, and individualized 7T networks could be a valuable future direction, but this is outside the scope of this work.

**Reviewer #3 (Public review):**

Summary:

This paper addresses an important question (the relationship between DN and dATN, and the role of retinotopic coding) and uses a set of novel analyses.

Strengths:

Important question, novel analytical approaches (pRF-informed functional connectivity analysis).

Weaknesses:

Some of the key claims are not fully supported by the data presented. There is also a concern about over-interpretation of the results. Key issues:

(1) The authors claim that retinotopic coding scaffolds the interaction between DMN and dATN. However, retinotopically tuned voxels account for a mere 9% of DMN voxels. So this appears to be a major overstatement. For instance, the statement that "these findings would position retinotopy as a unifying framework for brain-wide information processing" is not justified given the presented data.

We appreciate the reviewer’s concern about the framing of our conclusion, which was shared by reviewers 1 and 2. In response to these comments, we have revised our paper to more accurately reflect the observed data. Specifically, we focus on the specific sub-populations of voxels within the DN and dATN that show retinotopic responses, and we have removed references to explaining the overall pattern of activity across networks.

(2) Given that positive pRF voxels in DMN positively correlate with dATN voxels and negative pRF voxels in DMN negatively correlate with dATN voxels, there is a concern that these results could be contributed to by imprecise brain network parcellations. E.g., could some of the positive pRF voxels in DMN be erroneously assigned to DMN and actually belong to one of the other task-positive networks? There is insufficient validation of network parcellation to put this worry to rest, especially since it depends on ICA, which has a degree of arbitrariness built in.

We thank the reviewer for the opportunity to clarify our method for network definition.

Precision functional mapping is a growing field with many methods for defining personalized functional networks for each individual. Because the NSD resting-state data is relatively high resolution, we chose an approach designed to improve the stability of voxel-wise network assignment: Multi-Session Hierarchical Bayesian Modeling approach (Kong et al. 2019; Du et al. 2024). This approach enhances stability of network assignment by including a group-based prior and accounting for both within- and across-subject variability. This approach is more stable than ICA, and, because this approach leverages a prior, there is less concern about arbitrary or idiosyncratic network definitions.

However, it is still common for network assignments to have lower confidence around the borders between networks. Yet, we also do not think border misassignment is likely to explain the present results for two reasons: first, while DN and dATN nodes are sometimes adjacent, there are many regions where they are spatially distant, such as the IPS for dATN and the lateral temporal lobe for DN. Second, the DN pRFs do not appear to cluster selectively along DN–dATN borders, suggesting that they are not simply misassigned dATN voxels.(Fig. S3) Therefore, we think voxels on the edge of these networks are unlikely to drive the effects observed here (see Fig. S3).

(3) The claim that retinotopic coding is intrinsic to the DN network is not supported by rigorous analysis and results. The analysis here has many arbitrary factors, including: the threshold of the 99th percentile of resting-state distribution; the designation of DN as "top-down" and dATN as "bottom-up"; the definition of "anti-matched" voxels instead of using randomly selected voxels; and the statistics being paired between matched and anti-matched voxels instead of using comparisons to baseline. Overall, I do not think that the result supports the conclusion that retinotopic coding in DN is intrinsic instead of being bottomup-driven, given the very high threshold (99%) used and the fact that many other networks could also send bottom-up input to DN. Furthermore, the idea that bottom-up inputs only occur when the dATN (or any other RSN)'s spontaneous BOLD activity is above a certain threshold is a huge and unvalidated assumption.

The reviewer raises several interesting concerns about decisions in our event-detection analysis. Here, we clarify the rationale for several analytic choices:

(1) The 99th-percentile threshold was chosen to identify sparse, high-amplitude events while minimizing contamination from smaller ongoing fluctuations.

(2) The other reviewers also noted a concern with the top-down/bottom-up terminology. We have revised these terms to DN-driven and dATN-driven, which we think reflect our approach more accurately.

(3) We used anti-matched rather than randomly selected voxels because the full event-by-voxel randomization procedure was computationally intractable at the network level.

(4) We did not understand the reviewer’s contention about activation baseline, but we would welcome clarification.

**Recommendations for the authors:**

**Reviewer #1 (Recommendations for the authors):**

Minor points

(1) The reliability analysis (Figure 1D) notes that dATN negative pRF amplitude was not reliable above chance in 2 of 7 participants. This could be discussed more prominently as it suggests that negative pRFs may not be stable features in all networks, which tempers the generality of the sign distinction as a fundamental organizational property.

We thank the reviewer for raising this point of clarification. It is true that 2/7 participants did not show reliable negative pRFs in the dATN. However, the majority of participants show stable negative pRFs, and even in these 2 participants, the negative result does not indicate that negative pRFs would not be stable in those individuals with additional data.

Based on the reviewer’s comment, we have added emphasis to this point, but we do not feel that this warrants greater discussion in the paper.

Both positive and negative pRF amplitude was reliable in the DN for all subjects. In the dATN, positive amplitude pRFs were reliable in all participants, and negative amplitude pRFs (which constituted a small proportion of the overall pRFs in this network) were reliable in 5/7 participants. For the remainder of the paper, we only consider positive pRFs in the dATN. Importantly, pRF center position was highly reproducible across runs of pRF data in the dATN and DN in all subjects (Fig. 1D). Pg. 5

(2) The paper would benefit from situating the findings more explicitly within the cortical gradient framework (Margulies et al., 2016), which predicts that DN regions have maximally abstract, transmodal codes. The present findings complicate this view productively and deserve to be "situated" within that ongoing debate.

We agree that the gradient framework is interesting, and we have added discussion of Margulies to our paper. (Pg. 16)

Relatedly, the DN is considered a transmodal hub for cortical processing, where disparate sensory and motor processes converge (59, 75) The DN’s position at the cortical apex implies connections with and influence over unimodal cortical areas. However, the mechanism for liking unimodal and transmodal networks had been unknown. Prior work posited that sensory coding in transmodal areas might serve this function (31, 35), and our data provide direct empirical support for this account: specific visually-responsive voxels provide an input/output interface linking perceptual and memory systems. This complements work delineating specific affective and effective subregions within the DN that link the DN to other brain areas (76). Thus, while the DN may be “distant from input” (28), these data suggest that it is not disengaged from sensory processing.

(3) It would be informative to know whether the *proportion* of negative vs. positive pRFs differs between DN-A and DN-B, given their distinct functional roles.

Despite the functional specialization of DN-A and DN-B, and the slightly higher mean proportion of negative pRFs in DN-A (61% vs 56%), we found no statistically significant difference in the proportion of negative pRFs across the two networks (t(6) = 0.888, *p* = 0.409).

(4) Low N is inherent to the densely-sampled NSD design, and the within-subject consistency is a strength. Nevertheless, with 6 degrees of freedom, the precision of specific quantitative estimates (e.g., that 58.77% of DN pRFs are negative) is uncertain, and the authors should be cautious about the generalizability of these point estimates.

The reviewer raises a concern about the inclusion of specific levels of decimal place in our statistical reporting. We do not think that this is a major issue with the paper, but we are willing to change if the reviewer feels strongly.

**Reviewer #2 (Recommendations for the authors):**

(1) Figure 1C could use an explicit legend (I believe it is following the color convention from the bar plots in 1F?). Also, for consistency, it would be helpful to make all the colormaps in 1F correspond to the bars (i.e., change the dATN colormap to go white->green).

We thank the reviewer for this suggestion, and we have added explicit labels to Fig. 1C

(2) Providing a scatter plot, in which each dot is a voxel and the x and y axes are the pRF amplitude estimates in different runs, could help provide evidence that there are voxels with pRFs that have consistently negative amplitudes across runs. This would also go beyond the binary consistency analysis in Figure 1D to show that the magnitudes of the amplitude estimates are also consistent.

We thank the reviewer for this suggestion. We feel that the binary consistency conveys sufficient information. Because the analysis is done using pairwise correlation, how the scatter plot would reflect the three-way consistency is not clear.

(3) For understanding how the overall correlation between DN and dATN could be driven by voxel populations with opposing effects (e.g., Figure 2B), it would be useful to show how the +pRF and -pRF voxels compare to other voxels within the DN. For example, are these the voxels with the strongest negative and positive correlations with dATN, or are there many other DN voxels (among the 90% that do not have pRFs) that also have similarly-strong dATN correlations?

The reviewer offers a very interesting suggestion. Based on the reviewer’s suggestion, we have refocused our paper on the particular subpopulations of +/- pRFs in the DN, rather than on an explanation for the overall pattern of correlation between the DN and dATN. Because our revised framing focuses on the properties of these retinotopically defined voxel populations, rather than on explaining whole-network DN–dATN coupling, we have not added this additional analysis. We have revised the relevant text to avoid implying that these pRF subpopulations drive the overall network-level relationship.

(4) Initially, the baseline comparison pRFs for the matched pRFs are labeled "random" pRFs, which seems misleading; these are closer to "mismatched"/"anti-matched" pRFs since they are selected from the 1/3 that are farthest away. Then the comparison switched to using the anti-matched pRFs that are the 10 very farthest away, though I didn't understand the rationale that "the large number of pRFs made the random matching procedure impractical" - in what way is the number of pRFs larger in this analysis? Having a more consistent baseline (e.g., just using the 10 anti-matched pRFs the whole time) would be easier to interpret.

We thank the reviewer for this suggestion. We have compared the results between the randomly-sampled bottom ⅓ matched versus the 10 worst matched, and the pattern of results is identical (the effect is strongest in the 10 worst matched). Therefore, we include the bottom ⅓ matched in the main text as a more conservative test of this effect. We are happy to include this as a supplemental figure if the reviewer feels it is essential.

(5) In the past, I have only seen the terminology "bootstrapped" to refer to sampling with replacement from the data sample, producing samples/statistics that are centered on the observed data. Here (lines 704-708), the sampling is coming from the null distribution of randomly-chosen voxels, and therefore the term "bootstrapped" would not apply (and could just be replaced with "null").

We have revised this terminology in the manuscript.

**Reviewer #3 (Recommendations for the authors):**

(1) Abstract and Discussion should be significantly toned down. E.g., the claim that "These findings challenge the prevailing view of global DN-dATN antagonism" is not really supported by the data provided. The claim that "retinotopic coding underpins the dynamic coordination of perception and thought" is also unsupported by the presented data.

We have revised the manuscript in light of this comment.

(2) Line 233-235: The null statistical result cannot support the claim reached here. Correlation analysis or Bayesian statistics should be used.

We have revised the manuscript in light of this comment.

(3) Line 250-254: Comparison to baseline should be used, in addition to comparing matched and random voxels.

We agree that baseline comparisons can be useful in event-triggered analyses. However, for the pRF-matching analysis discussed here, the critical question is whether shared visual-field preference influences resting-state functional coupling between DN and dATN voxels. For this question, we believe that the appropriate baseline is the coupling observed for pRFs that do not share visual-field preferences. We therefore compare retinotopically matched pRFs to randomly matched pRFs drawn from the same networks.

(4) Line 271: "not" is missing.

We have revised the manuscript in light of this comment.
